# Assessment of Breastfeeding Practices During Febrile Illness Among Mothers of Infants: Insights From Urban Slums of Bhubaneswar

**DOI:** 10.7759/cureus.80506

**Published:** 2025-03-13

**Authors:** Priyanka Mohanty, Krishna Mishra, Sumelika Das, Spandan Mishra, Baishnabi Pattanaik

**Affiliations:** 1 Department of Community Medicine, Kalinga Institute of Medical Sciences, Bhubaneswar, IND

**Keywords:** breastfeeding, febrile, health-seeking behavior, perception, urban slums

## Abstract

Background and aim: Breastmilk contains all the essential nutrients for growth and immunological factors for the protection of the baby against various diseases, which should not be discontinued unless contraindicated or advised by the doctor under specific conditions. In urban slums, where literacy levels are low and awareness is limited, the lack of knowledge about the importance of continuing breastfeeding during minor febrile illnesses may act as a barrier to breastfeeding. Therefore, this study was conducted among mothers of infants living in urban slums. This study aimed to assess the perceptions of mothers with infants regarding breastfeeding and their practices during any febrile illness, either of themselves or their baby, to identify associated factors and evaluate their health-seeking behavior during such episodes.

Methods: A cross-sectional study was conducted among 100 consenting mothers of infants below one year in the field practice area of a medical college. The study participants were selected using a simple random sampling technique using a computer-generated random number table. Those satisfying the inclusion and exclusion criteria were included in the study. The data were collected through face-to-face interviews using a semi-structured, pre-tested questionnaire. Data were coded, entered in an Excel sheet (Redmond, WA: Microsoft Corp.), and analyzed using Epi Info software version 7.2.6.0 (Atlanta, GE: Centers for Disease Control and Prevention). Data were interpreted in frequencies and percentages; the association was tested by the chi-square test and F-test, wherever applicable, and a p-value of less than 0.05 was considered statistically significant.

Results: The mean age of mothers was 22±3.21 years. The awareness regarding the continuum of breastfeeding during febrile illness of either the mother and/or the baby was lacking in around 27 (27%) respondents; 4 (4%) mothers perceived that breastfeeding might transfer infection to the baby and discontinued it. The educational status of the mother and type of family were found to be statistically significantly associated with the discontinuation of breastfeeding (p=0.009 and 0.010, respectively). The health-seeking behavior was found to be good in around 94 (94%) mothers.

Conclusion: Around 73% of the mothers perceived that breastfeeding should be continued during febrile illness and around 20% of them thought that illness transfers the infection from the mother to the baby which resulted in discontinuation of the same in some. The type of family was one of the identified associated factors of breastfeeding where residents of nuclear families were more aware of breastfeeding than the residents of joint families. Health-seeking behavior was found to be within the first 48 hours among 94% of the study participants.

## Introduction

Breastfeeding is one of the most effective ways to ensure a child's health and survival. However, contrary to the recommendations by the World Health Organization (WHO) fewer than half of infants under six months of age are exclusively breastfed. Breastfed children perform better in intelligence tests, are less likely to be overweight or obese, and are less prone to develop diabetes later in life. Women who breastfeed also have a reduced risk of breast and ovarian cancers [1-3]. Exclusive breastfeeding reduces the risk of many morbidities like middle ear infections, respiratory tract infections, asthma, and gastrointestinal acute illnesses. It is also associated with a reduced risk of sudden infant death syndrome (SIDS), certain skin diseases like atopic dermatitis and eczema as well as metabolic diseases like diabetes. Moreover, it has been shown to reduce the risk of childhood leukemia [4]. The American Academy of Pediatrics (AAP) recommends exclusive breastfeeding for the first six months and continuing breastfeeding even after the introduction of solid foods, until at least one year of age or until both mother and baby agree to quit [5].

Despite having many advantages, the awareness regarding the importance of breastfeeding and its continuity is ambiguous. Data from the National Family Health Survey-5 (NFHS-5) indicate that Odisha has demonstrated commendable achievements in maternal and child health. Specifically, 68.5% of infants in the state are breastfed within the first hour of birth, a figure that exceeds the national average of 41.8% of infants being breastfed within one hour of birth. Odisha outperforms the national average in exclusive breastfeeding during the initial six months, recording a rate of 72.9% compared to India’s overall rate of 63.7% [2].

This study was planned in urban slums, which comprise a vulnerable population with a relatively low literacy rate and a lower standard of living, factors that may predispose individuals to maternal or childhood febrile illnesses. There is a paucity of literature on breastfeeding practices among mothers during any febrile illness from this part of eastern India. With the above background, this study was planned to be undertaken among mothers of infants residing in urban slums to understand their perceptions and practices regarding breastfeeding during any febrile illness of the mother and/or child, identify the associated factors affecting breastfeeding practices during such illnesses, and assess their health-seeking behavior for the same. Though the barriers to breastfeeding have been quite researched, this aspect has not been much researched in this part of the country.

This article was previously presented as an oral paper at the 68th Indian Public Health Association Conference (IPHACON) on March 16, 2024.

## Materials and methods

Study design, population, and setting

This was a community-based, cross-sectional study conducted in the urban slums under the field practice area of the department of community medicine of a tertiary care hospital in eastern India. The study was undertaken from June 2024 to August 2024. 

Inclusion and exclusion criteria

Consenting mothers over the age of 18 years with infants below one year of age and residing in the slum for more than one year were included. Mentally unstable or uncooperative mothers, those where the mother was not the primary caregiver, those with chronic medical illnesses, and those who were unavailable even after two home visits were excluded from the study.

Sample size and sampling technique

The calculated sample size was 96, but responses were obtained from 100 mothers. The reference for sample size calculation was taken from a similar study by Kamath et al., conducted in southern India, which considered the percentage of breastfeeding practices during cold and fever in babies as 94%, a significance level of 5%, a 95% confidence interval, and 80% power. The calculated sample size was 87 [6]. Considering a nonresponse rate of 10%, a sample size of 96 ≈ 100 was taken for the study. A simple random sampling technique was used to select the study participants by using a computer-generated random number table. The first house was the first number generated from the random number table and then each subsequent numbers were followed till the sample size was achieved.

Study tool

A semi-structured, pre-tested questionnaire with three parts was used to collect data. The first part comprised of questions on the socio-demographic profile of the family, the second part was used to assess the perceptions and practices regarding breastfeeding during any febrile illness of the mother or baby. The third part had questions on health-seeking behavior during febrile illness of the mother/child or both. The questionnaire was pilot-tested in another nearby slum and necessary changes were included. The questionnaire was translated into the local language (Odia) while interviewing and back-translated to English during analysis.

Data collection method

A list of all the eligible mothers with infants below one year was obtained from the Anganwadi Centre. The principal investigator and her team performed a face-to-face interview in the local language after receiving written informed consent from the respondents. Data regarding the febrile illness were collected from the day the youngest child was born to the mother till the date of the interview (<1 year), as the study participants were only mothers of infants. As the period of recall was limited to a maximum of up to one year, the chances of recall bias were less.

Data analysis

The collected data were coded, entered into a Excel sheet (Redmond, WA: Microsoft Corp.), and analyzed using Epi Info software version 7.2.6.0 (Atlanta, GE: Centers for Disease Control and Prevention). Data were interpreted as frequencies and percentages. The association was tested by the chi-square test and f-test, wherever applicable. A p-value less than 0.05 was considered statistically significant.

Ethical considerations

Clearance from the Institutional Ethics Committee, Kalinga Institute of Medical Sciences, Bhubaneswar, was obtained via letter no. KIIT/KIMS/1878/2024, and the study was conducted in accordance with the Indian Council of Medical Research (ICMR) ethical guidelines for conducting biomedical research. Written informed consent was obtained from all study participants after explaining the purpose of the study. Confidentiality was maintained throughout the study. Participants were informed in advance that they could leave the interview at any time and skip any questions they did not wish to answer.

In this study, breastfeeding practices were considered to be "good" if more than 63.7% of the study participants were practicing the same; 63.7% being the national average of exclusive breastfeeding as per NFHS-5. Similarly, health-seeking behavior was considered to be "good" when the family/parents sought medical help for the febrile child/mother within 48 hours of illness and "poor" if it was delayed beyond 48 hours or not sought at all.

## Results

A total of 100 responses were collected on the various aspects of breastfeeding during the febrile illness of a mother or infant. Majority of the mothers were in the age group of 18-25 years with a mean age of 22±3.21 years. A maximum of them, 66 (66.00%), were homemakers and did not contribute to the family income. Around 98.00% (98) of the mothers knew about the importance of exclusive breastfeeding (EBF) but only around 73.00% (73) of the mothers perceived that breastfeeding should be continued even when the baby or the mother has an episode of febrile illness. The majority (73.53%) of the mothers in the age group of 18-25 years believed that it should be continued. It was found that the educational status of the mother (p=0.009) and the type of family (0.010) were found to be statistically significantly associated with the mother’s perception regarding the continuation of breastfeeding during any febrile illness of self or the baby. The association of socio-demographic features with the breastfeeding practices of mothers during febrile illness of the mother and/or the infant has been depicted in Table 1.

**Table 1:** Association of socio-demographic variables with perception towards continuation of breastfeeding during febrile illness of the mother and/or the baby (n=100). ^*^Regarding the education fathers included in the study, none were illiterate.

Socio-demographic variables	Category (n)	Breastfeeding should be continued during febrile illness		p-Value	Chi-square value/f-value	95% CI
		Yes (total=73), n (%)	No (total=27) n (%)			
Age of mother (in years)	18-25 (68)	50 (73.53%)	18 (26.47%)	p=0.862	0.030	0.78, 0.92
	26-45 (32)	23 (71.87%)	9 (28.13%)			
Age of spouse (in years)	18-25 (20)	16 (80.00%)	4 (20.00%)	p=0.610	0.26	0.51, 0.70
	26-45 (80)	57 (71.25%)	23 (28.75%)			
Age of baby (in months)	<1 month (5)	4 (80.00%)	1 (20.00%)	p=0.923	0.160	0.85-0.96
	2-6 months (72)	52 (72.22%)	20 (27.78%)			
	6 months-1 year (23)	17 (73.91%)	6 (26.09%)			
Birth order	First born (92)	68 (73.91%)	24 (26.09%)	p=0.777	0.080	0.69-0.85
	Second born (8)	5 (62.50%)	3 (37.50%)			
No of siblings	None (92)	68 (73.91%)	24 (26.09%)	p=0.777	0.080	0.69-0.85
	1-3 (8)	5 (62.50%)	3 (37.50%)			
Education of mother	Uneducated (29)	17 (58.62%)	12 (41.38%)	p=0.009	9.250	0.0015, 0.053
	Primary (46)	41 (89.13%)	5 (10.87%)			
	Secondary (15)	10 (66.67%)	5 (33.33%)			
	Graduate (10)	5 (50.00%)	5 (50.00%)			
Education of father*	Primary (64)	48 (75.00%)	16 (25.00%)	p=0.806	0.430	0.72, 0.87
	Secondary (24)	17 (70.83%)	7 (29.17%)			
	Graduate (12)	8 (66.67%)	4 (33.33%)			
Type of family	Nuclear (84)	66 (78.57%)	18 (21.43%)	p=0.010	6.600	0.0018, 0.054
	Joint (16)	7 (43.75%)	9 (56.25%)			

The perceptions of all the mothers were noted, and the practices were recorded only for the mothers who had an episode of febrile illness after the birth of their baby (n=83). There were no missing data. Around 83 (83.00%) mothers had reported having an episode of febrile illness after the birth of the youngest child. Around 73 (73.00%) mothers believed that they should continue breastfeeding and 21 (21.00%) were not sure about what they should do if they had an episode of febrile illness due to any reason. Around six (6.00%) believed that they should not breastfeed their child during any febrile illness. For the purpose of analysis, the "don’t know" was clubbed with "No." Around 20 (20.00%) mothers believed that maternal infections are transferred to the baby on feeding and seven (7.00%) of them perceived that breast milk secretion decreases during febrile illness of the mother. As many as 13 (13.00%) respondents did not know whether a mother taking any medication can/should continue feeding her infant as usual. Out of the 83 (83.00%) mothers who had a febrile episode, 57 (68.67%) continued feeding as usual and 71 (85.54%) fed as per doctor’s advice. The perceptions and practices of the mothers regarding breastfeeding during an episode of febrile illness have been represented in Table 2.

**Table 2:** Perception and practices of the mothers regarding breastfeeding during an episode of febrile illness (n=100). *Though the total number of study participants was 100, only 83 reported an episode of febrile illness after the birth of their youngest child, and responses on practices were received only from them.

Perception of mothers about breastfeeding during febrile illness	Frequency in n (%)
Mother should breastfeed her baby during her illness	
Yes	73 (73.00%)
No	6 (6.00%)
Don’t know	21 (21.00%)
Infection transfers from mother to baby through breast milk	
Yes	20 (20.00%)
No	80 (80.00%)
Breast milk secretion decreases during febrile illness	
Yes	7 (7.00%)
No	93 (93.00%)
Mother suffering from febrile illness and taking medication can breastfeed her baby	
Yes	78 (78.00%)
No	9 (9.00%)
Don’t know	13 (13.00%)
Frequency of breastfeeding should decrease when mother is febrile	
Yes	75 (75.00%)
No	25 (25.00%)
Breastfeeding practices by the mothers during their febrile illness (n=83)*	
Continue feeding as usual	57 (68.67%)
Expressed breast milk	11 (13.26%)
Milk substitutes	9 (10.84%)
Palladia	6 (7.23%)
Feed as per doctor’s advice	
Yes	71 (85.54%)
No	12 (14.46%)

**Figure 1:**
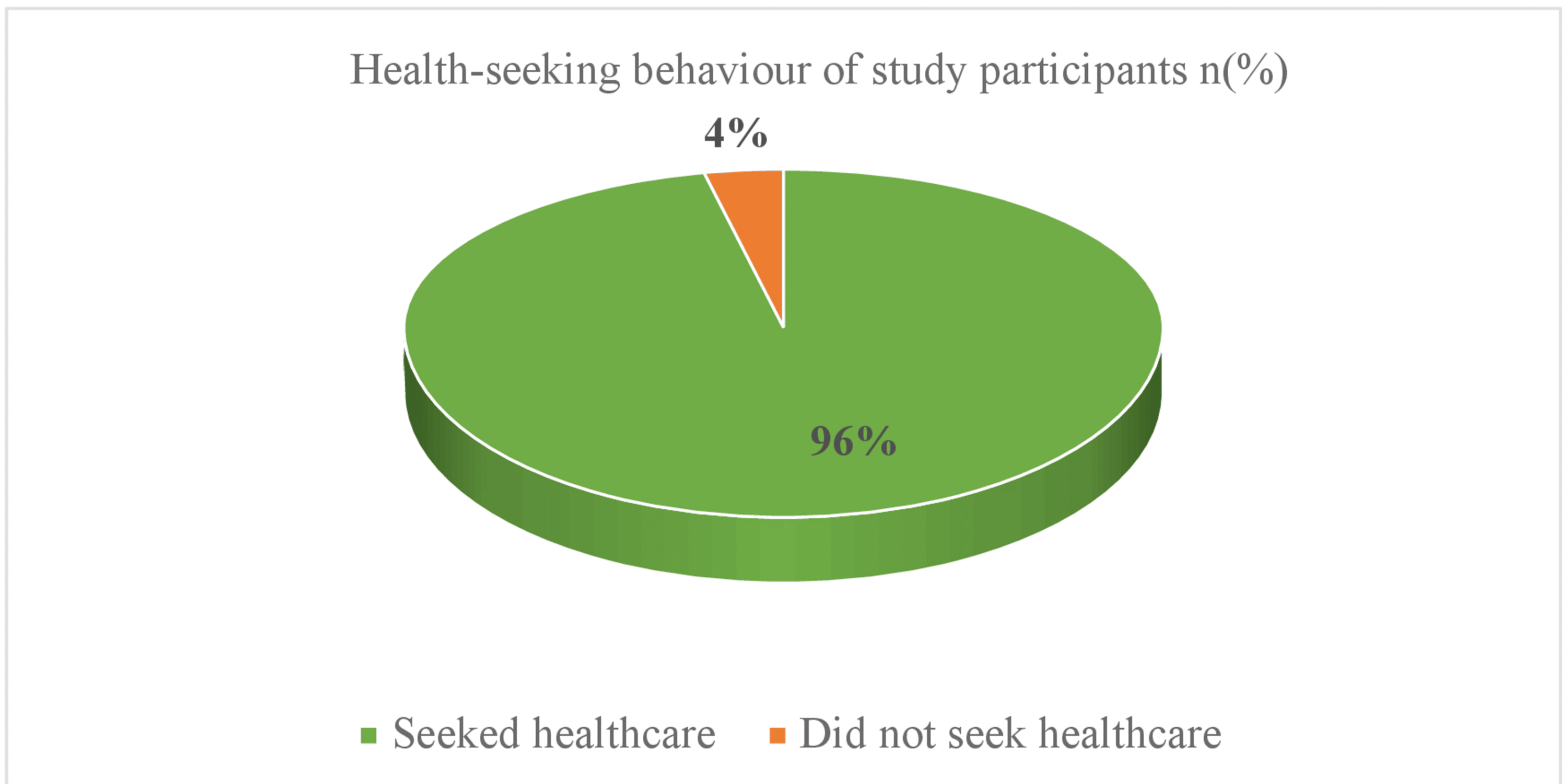
Health-seeking behavior of the mother based on their perception (n=100).

Around five (5.00%) mothers responded that the family members opposed breastfeeding the baby when the mother was unwell due to any reason. Around four (4.00%) mothers responded that their breastfeeding habit was altered based on some cultural practices like keeping the baby away when the mother was menstruating, feeding the baby with goat’s milk when mother was ill, and feeding the baby with milk substitutes or cow’s milk when the mother was on medication. One of the mothers believed that the baby should only be cured with traditional medications like the use of herbs instead of prescribed medications by the doctor with the belief that it could harm the gut of the baby. There was no statistically significant association between maternal perception regarding breastfeeding during febrile illness and health-seeking behavior regarding the same (p=0.062). It was seen that around 94 (94.00%) mothers either had or would prefer to seek healthcare during a febrile illness of self or baby whereas six (6.00%) responded that they would wait for two days or more and try home remedies before seeking healthcare for self or the baby. The health-seeking behavior of the mothers during febrile illness of self or the baby has been represented in Figure 1.

**Figure 2:**
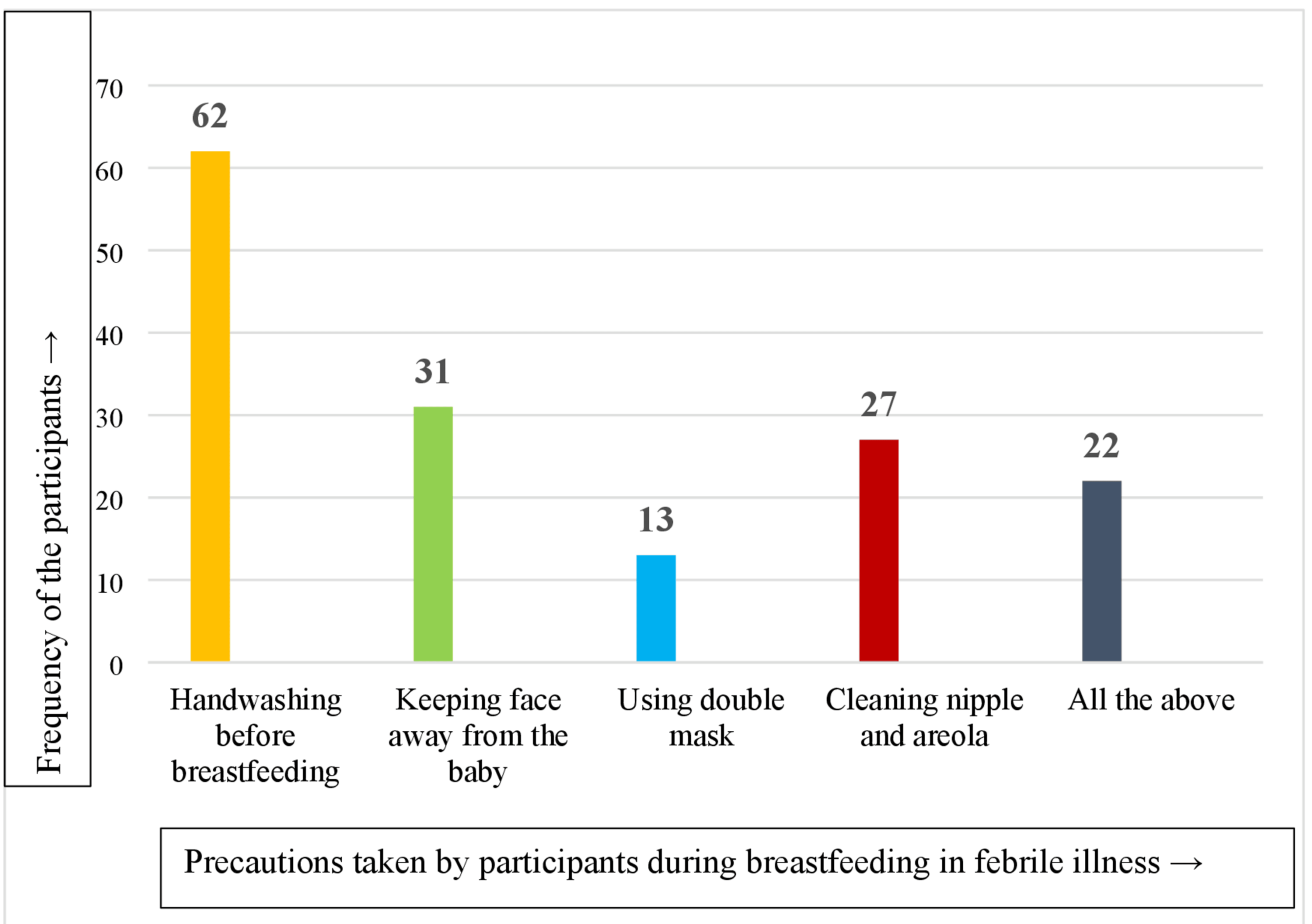
Responses of the study participants about the precautions to be taken while breast feeding during febrile illness (multiple responses).

Majority of the mothers knew that they should wash their hands before breastfeeding during any illness. Around 22 (22.00%) mothers perceived that they should practice all the mentioned precautions while breastfeeding the baby when they were ill. Some of the mothers had multiple responses for the precautions to be taken. Figure 2 shows the precautions mothers believed should be taken while breastfeeding during a febrile illness.

## Discussion

The current study aimed at assessing the breastfeeding practices among 100 consenting mothers of infants residing in urban slums of Bhubaneswar, Odisha; where most of the mothers were in the age group of 18-25 years with a mean age of 22±3.21 years. The majority of mothers were Hindus, most of them were educated till primary school 46 (46.00%), most of them were housewives and majority were residents of nuclear families, 84 (84.00%). In a study done by Davra et al. in Vadodara on lactating mothers, the mean age of the mothers was 24.9±3.8 years [7]. This finding is higher than that of the present study as the age of marriage is usually early in slums. Other findings of this study are similar to the cited study including majority of mothers being Hindus, educated at least till primary school, and most of them being housewives by occupation. In Davra et al.'s study, most of the study participants belonged to joint families, whereas in the present study majority were residents of nuclear families [7]. The difference in the type of family could be due to differences in social and cultural norms. The factors like education of the mother and type of family were found to be significantly associated with the perception that breastfeeding should be continued during an episode of febrile illness in the present study, and this finding differs from the findings of the above-mentioned study.

In this study, 83 (83.00%) participants reported that they had at least one episode of febrile illness post-delivery, and out of them around 57 (68.67%) continued feeding as usual, and 71 (85.54%) fed as per doctor’s advice. A study conducted by Libraty et al. in the Philippines to evaluate the preventive effect of breastfeeding on infants with febrile illness reported that infants who were breastfed had lower odds (0.57 {0.34-0.94}) of being affected by febrile illness [8]. This emphasizes the need for a continuum of breastfeeding. In this study, 57 mothers (68.67%) continued to breastfeed as usual during illness. In contrast, a study by Kamath et al., conducted at a district tertiary government hospital in southern India, found that approximately 79.70% of mothers were knowledgeable about the importance of breastfeeding. Furthermore, 94.00% of them believed that breastfeeding should be continued during a baby's febrile illness, and 90.00% thought it should be continued if the mother was ill [6]. These findings are better than the findings of the current study. This difference might be because of the difference in the study setting. In a study done by Nguyen et al. in Vietnam, some mothers perceived that it was difficult for them to breastfeed when the baby was ill [9]. However, the maternal educational level was found to be significantly associated with the perception of continuation of breastfeeding in infants during any febrile illness in both studies. In this study, 94 (94.00%) mothers showed good health-care-seeking behavior. A study done in Zambia by Apuleni et al. reported that maternal education was one of the important predictors of good health-seeking behavior among breastfeeding mothers during illness of their baby [10]. In the present study, maternal education was one of the determinants of breastfeeding practices during febrile illness. In a study done in Nigeria on health-seeking behavior among mothers of under-five children by Nweya et al., it was reported that only 72.9% of the mothers had good health-seeking behavior during any childhood illness [11]. The present study reported that the health-seeking behavior of the participants was better than the above-mentioned study.

In the present study, only two mothers (2.00%) did not exclusively breastfeed until six months, and a few of them gave goat milk as pre-lacteal feed. In contrast, a study by Meharda and Dixit, conducted in the Phagi district of Jaipur, Rajasthan, found that around 67.33% of children were not exclusively breastfed, and 62.66% of infants were given pre-lacteal feeds [12]. In another study done by Rajak et al., pre-lacteal feed (mostly honey) was given to 33% of the newborns [13]. The findings of the current study are better than both the studies cited above and this difference might be due to the difference in the study settings and cultural beliefs affecting the perception and practices of the breastfeeding mothers. Around 22 mothers (22.00%) in the present study took all the precautions, such as handwashing, cleaning the nipples and areola, wearing a face mask, and/or keeping their face away from the baby during breastfeeding when experiencing a febrile illness. In contrast, a study by Kaushik and John, conducted in a selected community in Delhi, found that 31.60% of mothers had a good practice score with respect to breastfeeding and breast hygiene in general [14]. There are many studies regarding awareness, importance, benefits of breastfeeding, and the barriers to continuing the same but there are not many published studies about this topic, particularly about breastfeeding practices of mothers during any febrile illness of the mother or the baby [15-18].

Targeted intervention in the form of health education was given to the study participants of the present study to address the identified gaps. However, it is recommended that social support for breastfeeding should be emphasized to ensure continuity of breastfeeding.

Limitations

Being cross-sectional in nature, causality cannot be established. The results of the study cannot be generalized as the current study has been conducted among urban slum resident mothers.

## Conclusions

The number of mothers continuing breastfeeding during an episode of a febrile illness among slum residents was found to be 73%. There were certain determinants that were identified to be associated with the continuation of breastfeeding among the study population during an episode of febrile illness. The health-seeking behavior was found to be good among the resident mothers in urban slums.

With a continuous effort to create awareness regarding the various aspects of breastfeeding and its importance which is already a practice of the institute, it is possible to overcome the identified gaps to strengthen maternal and child health; thereby improving child survival. The health education regarding the precautions to be taken during breastfeeding when the mother is ill has been provided to all participant mothers to avoid discontinuation of breastfeeding unless advised by a doctor. However, this study identified that demographic factors, such as maternal education, were among the factors affecting breastfeeding practices. Therefore, targeted educational sessions for mothers with low literacy could be helpful in addressing the identified gap. Family type was also one of the determinants of continuation of breastfeeding during febrile illness, where mothers in nuclear families had better practices than those in joint families. This re-emphasizes the need for family and social support for sustaining breastfeeding. More research is required for the assessment of proper breastfeeding practices during other ailments. Studies involving strategies to ensure behavior change in this aspect are recommended to improve breastfeeding practices and achieve the national target of ensuring exclusive breastfeeding of 90%.
